# Parasuicide and drug self-poisoning: analysis of the epidemiological and clinical variables of the patients admitted to the Poisoning Treatment Centre (CAV), Niguarda General Hospital, Milan

**DOI:** 10.1186/1745-0179-1-5

**Published:** 2005-04-28

**Authors:** Massimo Carlo Mauri, Giancarlo Cerveri, Lucia Sara Volonteri, Alessio Fiorentini, Alessandro Colasanti, Sergio Manfré, Rossana Borghini, Emma Pannacciulli

**Affiliations:** 1 Clinical Psychiatry, University of Milan, Clinical Neuropsychopharmacology Unit, IRCCS Ospedale Maggiore of Milan, Italy; 2 Poisoning Treatment Centre (CAV) Ospedale Niguarda Ca' Granda of Milan, Italy

**Keywords:** parasuicide, drug self-poisoning, psychiatric diagnoses, risk factors

## Abstract

Epidemiological knowledge of parasuicides and drug self-poisoning is still limited by a lack of data. A number of preliminary studies, which require further analysis, evidenced that parasuicidal acts occur more often among females, that the peak rate is generally recorded between the ages of 15 and 34 years and psychotropic medications seems to be the most frequently used. The aim of this study was to describe the demographic and clinical variables of a sample of subjects admitted to the Posisoning Treatment Centre (CAV), Niguarda General Hospital, Milan, following drug self-poisoning. Furthermore, this study is aimed to identify the risk factors associated to parasuicidal gestures, with special care for the used drugs, the presence of psychiatric or organic disorders, alcoholism and drug addiction.

The study included the 201 patients attending the CAV in 1999 and 2000 who satisfied the criteria of self-poisoning attempts: 106 cases in 1999 and 95 in 2000.

The sample had a prevalence of females (64%). The peak rates of parasuicides from drug self-poisoning were reached between 21 and 30 years among the females, and 31 and 40 years among the males. 81.6% of the patients used one or more psychoactive drugs, the most frequent being the benzodiazepines (58.7%), classic neuroleptics (16.9%) and new-generation antidepressants (SSRIs, SNRIs, NARIs) (12.9%). The prevalence of mood disorders was higher among females (64% *vs *42%), whereas schizophrenia was more frequently diagnosed in males (22% *vs *10%). 61% (33%) had a history of previous attempted suicides. The presence of clinically relevant organic diseases was observed in 24.9% of the sample.

## Introduction

From the point of view of terminology, European suicidology seems to be oriented towards defining parasuicide as all non-fatal suicidal behaviours, regardless of their intentional nature (which is often difficult to investigate *a posteriori*). In other words, parasuicide covers behaviours that may vary from an exclusively manipulatory attempt, to an intentionally suicidal gesture and a serious act that was not fatal by chance (including attempted and failed suicide).

However, the current difficulty in unequivocally defining and officially classifying suicidal behaviours (suicide, attempted suicide, failed suicide, parasuicide) is demonstrated by the substantial absence of specific diagnostic criteria in the most widely used diagnostic and epidemiological manuals. The DSM [1] manual limits itself to including self-harming gestures among the diagnostic criteria of other psychiatric categories (major depression, borderline personality disorders), while ICD [2] classification is more specific and speaks of suicide and self-inflicted injury, including injures in suicide and attempted suicide, and self-inflicted injury specified as intentional.

The incidence of parasuicidal behaviours is ubiquitously higher among females, with a female/male ratio of between 0.71:1 and 2.15:1, with a median of 1.5:1 [3]. In a study aimed at verifying the trend of parasuicides from drug self-poisoning in 1987–1988 and 1992–1993, Bialas et al.[4] confirmed the prevalence of such behaviours among females and a female/male ratio of 1.13:1.

The peak rate of parasuicidal acts is recorded between the ages of 15 and 34 years, with the minimum incidence being reached after 55 years [3,5]. Similarly, the incidence of drug self-poisoning attempts peaks at the age of 15–25 years [4,6].

At least half of the patients making suicidal gestures do so using prescription drugs [7]. The must frequently involved are psychotropic medications, which are used in 80% of the cases of fatal deliberate self-poisoning and 68% of parasuicides [8,9]. Over the last few decades, there has been a considerable change in the types of drugs more frequently responsible for self-poisoning: barbiturates were the principal cause in the 1960s but, since the 1980s, the benzodiazepines have been the most frequently involved [10]. The data of Mc Loone and Crombie [6] indicate an increase in the number of cases of paracetamol intoxication for parasuicidal purposes as from the 1980s, followed by analgesics, antirheumatics, antedepressants and antipsychotics. These data were confirmed by Bialas et al. [4], who found that paracetamol was used for parasuicidal acts in 43.3% of the cases in 1992–1993, as against 31.3% in 1987–1988. The involvement of antidepressants increased from 11.3% in 1987–1988 to 17.6% in 1992–1993 [4], whereas there has been a trend towards a decrease in the parasuicidal use of benzodiazepines, particularly among women [6,11].

The data of Alsen et al. [9] indicate that there is no significant difference in the distribution of the drugs used for parasuicide and completed suicide but, according to Michel et al. [8], only barbiturates are significantly associated with fatal self-poisoning, whereas there is no significant difference in the case of tricyclic antidepressants, which are involved in 13% of deaths due to self-poisoning and 10% of parasuicidal acts [8].

The estimated annual incidence of parasuicides in Europe is between 300 and 800/100,000 inhabitants aged more than 15 years, with significant inter-country differences [12]. Meehan et al. [13] suggest that more than 75% of non-fatal suicidal gestures are not included in the official figures. Current knowledge of the real incidence of parasuicides in Italy is particularly limited by the lack of official statistics, which almost exclusively relate to fatal cases.

Parasuicidal behaviour is one of the most significant risk factors for death due to suicide, given that 30–60% of such deaths are the outcome of a series of characteristically repeated attempts [14,15].

The aim of this study was to describe the demographic and clinical variables of a sample of subjects admitted to the Poisoning Treatment Centre (CAV), Niguarda Hospital, Milan, following drug self-poisoning during the years 1999 and 2000. In addition to contributing to the epidemiological definition of parasuicide in our area, it also had the aim of identifying the risk factors correlated with such behaviours.

## Materials and methods

The study included all of the patients attending the Emergency Department, Niguarda Hospital, Milan, in 1999 and 2000 who satisfied the following criteria:

• A diagnosis of self-inflicted poisoning by drugs according to the ICD-9 criteria [2]

• A stay of at least one day in the hospital's Poisoning Treatment Centre (CAV)

Two hundred and one subjects satisfied these criteria (106 in 1999 and 95 in 2000), all of whom came from the Province of Milan, whose resident population at that time was 3,773,893 inhabitants with a slight prevalence of females (51.7%) [16].

### Evaluation of the study subjects

At the time of admission, all of the patients underwent clinical and toxicological evaluations.

Clinical evaluation:

• Physical examination

• Hemochromocytometric examination with leukocytic formula

• Markers of renal and hepatic function

• Plasma electrolytes, glycemia and cholesterolemia

• Arterial pressure and ECG monitoring

Toxicological evaluation:

• Plasma levels of the taken drugs

• Plasma levels of substances of abuse, if any

• Alcoholemia: it needs to be specified that alcohol intoxication at the time of the gesture was assessed exclusively on the basis of the presence of alcoholemia regardless of plasma alcohol levels because the extreme variability in the time between the parasuicidal gesture and hospital admission makes it difficult to establish the amount of alcohol actually consumed.

After the stabilization of their medical condition, all of the patients underwent a psychiatric evaluation during which the specialist collected the following information:

• The circumstances related to the parasuicide

• Previous parasuicidal episodes

• Previous contacts with psychiatric services

• Current and previous history of drug and alcohol abuse

• Concomitant organic diseases having a clinically relevant psychopathological impact or affecting life expectancy (cardiovascular disturbances, oncological diseases, acquired immunodeficiency, diabetes mellitus).

When appropriate, at the end of the clinical interview, the psychiatrist formulated a psychiatric diagnosis on the basis of the DSM IV criteria [1].

The data were statistically analyzed descriptively, using Student's T test to compare continuous variables and the chi-squared test to compare discrete variables.

## Results

The selected sample consisted of 201 patients: 73 males (36%) and 128 females (64%) with a mean age of 40 years (± SD 15.29; range 17–89).

The collected data indicate that parasuicides from drug self-poisoning accounted for 59.8% of the total of 336 CAV admissions in the years 1999–2000 (CAV database).

The mean time between the committal of the parasuicidal act and Emergency Department admission was 4.8 hours ± SD 6.03 (range 0.5–48.0).

### Drugs taken

In making their parasuicidal act, 118 subjects (58.7%) used at least one benzodiazepine, 34 (16.9%) at least one classic neuroleptic, 9 (4.4%) at least one atypical antipsychotic agent, 26 (12.9%) at least one second-generation antidepressant (SSRI, SNRI, NASSA), 11 (5.4%) at least one tricyclic antidepressant, 12 (5.9%) at least one mood stabilizer, 4 (1.9%) at least one antiepileptic drug (barbiturates), 21 (10.4%) at least one non steroidal anti-inflammatory drug (NSAID), 15 (7.4%) at least one antihypertensive agent, 8 (3.9%) at least one antispastic drug, 6 (2.9%) at least one antiallergic drug, 4 (1.9%) at least one antibiotic, 3 (1.4%) at least one diuretic, 4 (1.9%) at least one hypoglycemic agent, 2 (0.9%) at least one antiemetic, 2 (0.9%) at least one antiarrhythmic agent and 11 (5.4%) drugs with other indications [Table 1].

**Table 1:** Drug classes used for parasuicide purposes and their relative frequency of use.

Pharmacological class	No. of subjects	% frequency
Benzodiazepines	118	58.7
Classic neuroleptics	34	16.9
Atypical antipsychotics	9	4.4
SSRIs, SNRIs, NASSAs	26	12.9
TCAs	11	5.4
Mood stabilizers	12	5.9
Anticholinergics	4	1.9
Antiepileptics	4	1.9
NSAIDs	21	10.4
Antihypertensives	15	7.4
Antispastics, prokinetic antidiarrhoics	8	3.9
Antiallergics	6	2.9
Antibiotics	4	1.9
Diuretics	3	1.4
Hypoglycemic agents	4	1.9
Antiemetics	2	0.9
Antiarrhythmics	2	0.9
Alcohol	23	11.4
Substances of abuse and methadone	3	1.4
Miscellaneous	11	5.4

SSRIs: Serotonin Selective Re-uptake inhibitors; SNRIs: Serotinin Noradrenaline Re-uptake Inhibitors; NASSAs: Noradrenaline Selective Serotonin Antidepressants; TCAs: Tricyclic Antidepressants; NSAIDs: Non Steroidal Anti-Inflammatory Drugs

Twenty-three subjects (11.4%) had consumed alcohol at the time of their parasuicidal act, but in combination with drugs in all cases, and three (1.4%) had taken a substance of abuse (cocaine, heroin) or methadone, in all cases in combination with drugs for different indications [Table 1].

### Gender

The study population included significantly more females than males (64% *vs *36%), but their mean age was not significantly different (females 39 years ± SD 14.69 vs males 41 years ± SD 16.31).

In comparison with the population of origin (subjects resident in the Province of Milan in that period), the proportion of females in the study sample was significantly higher (51% vs 64%; chi-square = 11.125; p < 0.001) [16].

Age-group analysis revealed that the incidence of parasuicides peaked between the ages of 21 and 30 years among the females (32% of the sample) and between 31 and 40 years among the males (40% of the sample). Comparison of the age distribution of the residents in the Province of Milan with that of the study population showed that females aged 21–30 years were over-represented in the latter (32% vs 13%), as well as males aged 31–40 years (40%vs 18%) [16].

The results of the analysis of clinical variables by gender were as follows [Table 2]:

**Table 2:** Clinical-anamnestic variables by gender.

	Males (%)	Females (%)	Significance
History of parasuicide	30.8	30.4	No Significance
No history of parasuicide	69.2	69.6	

Drug addiction	16.4	4.0	Chi-square = 10.423
Alcoholism	19.2	15.7	p = 0.005
No history of dependence	64.4	80.3	

Mood disorders	42.0	64.6	Chi-square = 8.347
Anxiety disorders	6.0	1.3	p = 0.039
Schizophrenia	22.0	10.1	
Other	30.0	24.0	

• Despite the prevalence of females in the study population, there was no significant difference between gender in terms of a history of previous parasuicidal gestures (about 30% in both groups).

• The frequency of alcoholism and drug addiction was significantly lower among the females (p = 0.005).

• The distribution of psychiatric diseases was significantly different in the two groups, with mood disorders being more prevalent among females (64% *vs *42%) and schizophrenia more prevalent among males (22% *vs *10%) (p = 0.039).

### Sociodemographic variables

The sample consisted of 169 Italians (85%), six citizens of the EU (3%) and 24 citizens of non-EU countries (12%).

Sixty percent of the subjects were unmarried; the remaining 40% were married or co-habiting. In terms of occupation, 55% of the sample were students or employed, 16% unemployed, 21% pensioners, and 8% housewives.

### Clinical characteristics

Sixty-one subjects (30%) had a history of previous parasuicidal acts.

Analysis of the clinical symptoms present at the time of admission to the Emergency Department showed that 24 subjects (12%) were free of symptoms, 153 (76%) had symptoms attributable to central nervous system (CNS) depression (psychomotor slowing, drowsiness) and 24 (12%) had symptons of CNS activation (anxiety, psychomotor agitation).

At the time of admission, 40 subjects (20%) had altered laboratory test results (hemochrome with leukocytic formula, hepatic and renal function markers, plasma electrolytes, glycemia, cholesterolemia) and 23 (11.5%) electrocardiographic alterations.

#### Psychiatric diagnoses

Sixty-nine subjects (34%) did not have a history of psychiatric disorders. Of the other hand 132 (66%) subjects had a previous psychiatric diagnosis: 72 (35%) had a previous diagnosis of mood disorder, 30 (15%) of personality disorders and 19 (9%) of schizophrenia; the remaining cases (7%) had other psychiatric diagnoses, including anxiety and eating behaviour disorders.

The mean age of the subjects without a previous psychiatric diagnosis was significantly lower (35.98 years ± SD 14.54 *vs *42.03 years ± SD 15.32; t = 2.724; p = 0.007).

Among the subjects with a previous psychiatric diagnosis, those affected by personality disorders (30) had a mean age of 37.00 years ± SD 13.57, whereas the mean age of the other 102 was significantly higher (43.52 years ± SD 15.54; t = 2.235; p = 0.030).

The frequency of previous parasuicidal acts was significantly higher among the patients with a previous psychiatric diagnosis (45% *vs *11.7%; Chi-square = 19.837; p = 0.000).

#### Alcoholism and drug addiction

Thirty-five subjects (17.5%) had a diagnostic history of alcoholism, and seven (8.5%) a diagnostic history of substance abuse.

The time between the parasuicidal gesture and admission to the Emergency Department was significantly shorter for the alcohol abusers than the non-alcohol abusers (2.6 hours ± SD 3.8 *vs *4.9 hours ± SD 6.4; t = 2.772; p = 0.007).

#### Organic diseases

Fifty subjects (24.9%) had clinically relevant organic diseases (HIV, oncological or cardiovascular diseases).

In comparison with the rest of the sample, these subjects were significantly older (49.40 years ± SD 16.66 *vs *36.84 years ± SD 13.46; t = 4.825; p = 0.000) and had previously a significantly higher number of parasuicidal gestures (46.5% *vs *29.9%; chi-square = 4.018; p = 0.045).

## Discussion

The results of this study indicate that parasuicidal acts involving drugs accounted for a considerable proportion (56.8%) of the admissions to CAV in the years 1999–2000. Furthermore, these cases represented the majority of the self-inflicetd poisoning received by the Centre (the rest of the cases were mainly due to accidental intoxication caused by carbon monoxide, phosphorus poisoning, detergents or the dietary intake of toxic agents). Nevertheless, it is necessary to stress the difficulty in evaluating *a posteriori *the correlation between these cases and real suicidal intentions.

### Drugs taken

The published data agree in indicating that the drugs most frequently used for parasuicidal acts are those used to treat psychiatric disorders [5,9]. In line with these observations, 164 subjects in our sample (81.6%) used one or more psychoactive drugs, including 48 subjects (corresponding to 23.9% of the sample) who combined them with other pharmaceutical specialties. The most frequently used drug classes were the benzodiazepines, which were involved in the parasuicide of 118 patients (58.7% of the sample), followed by classic neuroleptics (16.9%), second-generation antidepressants (12.9%) and NSAIDs (10.4%). These findings confirm the previously observed large-scale use of the benzodiazepines for parasuicidal purposes [9,17], and can be attributed to their widespread prescription and ready availability. However, the low fatality index of the benzodiazepines should not lead us to underestimate the self-harming intentionality of parasuicidal gestures because there may be little relationship between the two, except perhaps in the case of self-poisoning on the part of healthcare workers, who are aware of the degree of lethality of the means used. Furthermore, benzodiazepines are also the most frequently used drugs in cases of fatal suicidal gestures [9]. It is likely that the drugs used to make the parasuicidal gesture are those related the current or previous therapy of the underlying psychiatric disorder. This is confirmed by the fact that 132 subjects (66% of the sample) had a history of a previous psychiatric diagnosis for which they probably received a pharmacological prescription.

Our data do not agree with some recent findings indicating NSAIDs (particularly paracetamol) as drugs frequently used for parasuicidal purposes [4,6,18].

A majority of the sample (114 subjects, 56.7%) combined two or more drugs in making their parasuicidal gesture, probably seeking a cumulative effect.

The parasuicidal gesture of 63 subjects (31.4%) was related to the improper use of one or more drugs intended for the treatment of organic diseases, including 28 (13.9% of the sample) who used them in combination with psychoactive drugs. This finding is consistent with the fact that 24.9% of our sample were affected by organic diseases.

### Gender

Women accounted for 64% of our study sample, which is in line with the findings of numerous studies confirming the prevalence of females in the ambit of parasuicidal behaviours [19-22]. Some authors believe that the higher prevalence of females is due to the search for means having less corporeal impact, regardless of whether the intent is really suicidal or not [5,23].

In line with the findings of Batt et al. [22], our results indicate that females are at peak parasuicidal risk at a younger age than males (21–30 *vs *31–40 years).

Among the females, the prevalence of alcoholism and drug addiction was significantly less than among men. The distribution of psychiatric diseases was also different between the two groups: females had a higher prevalence of mood disturbances (64% *vs *42%) and males a higher prevalence of schizophrenia (22% *vs *10%).

### Sociodemographic variables

Non-Italians accounted for 16% of our sample: 3% EU and 13% non-EU citizens. The official ISTAT census figures relating to the same period indicate that only 4.6% of the residents in the Province of Milan are not Italians, a difference that is probably related to an underestimate of the real number of foreigners in the Province, as well as to a greater risk of parasuicide among foreigners due to difficulties of adapting to the new environmental context.

The high percentages of unmarried (60%) and unemployed subjects (16%) is in line with published data indicating these two conditions as risk factors for parasuicidal gestures [24,25].

### Clinical characteristics

Twenty-five percent of the sample were affected by severe organic diseases, 66% by psychiatric diseases, and 26% by alcoholism or drug addiction. These data describe a population characterised by considerable somatic, psychic and probably social difficulties.

Furthermore, 30% of the cases had a history of previous parasuicidal episodes, thus confirming the substantial to repeat such gestures [15]. Kreitman and Casey [26] point out that only 40–60% of the subjects receiving medical attention after a parasuicidal gesture do not have a previous history of such episodes. The dramatic nature of the acute presentation of the majority of parasuicidal gestures tends to distract attention from the repetitive pattern of these behaviours, but the recognition that they may be one of a series in a significant number of cases implies the need to extend patient monitoring considerably beyond the acute condition.

#### Psychiatric diagnoses

The majority (66%) of our patients had a history of a previously diagnosed psychiatric disorder: a mood disorder in 35% of cases, a personality disorder in 15%, schizophrenia in 9%, and others (including anxiety and eating behaviour disorders) in the remaining 7%.

These findings are in line with the published data, according to which 70–90% of the subjects making parasuicidal gestures have a history of repeated contacts with psychiatric services [5,21,27].

Several studies [28-30] found that mood disorders, followed by disorders due to substance abuse, are the psychiatric diagnoses most frequently associated with suicidal and parasuicidal behaviours.

However, a number of recent clinical observations suggest that the absolute preponderance attributed to these disorders in relation to the risk of suicide needs to be reviewed [31,32].

Much of the published data agree that schizophrenia (particularly its paranoid form) is associated with a high risk of suicide [33,34], and 9% of the patients in our sample were schizophrenic. Given that recent definitions of post-psychotic depression underline the presence of a subsequent affective disorder arising during the course of schizophrenia [35-37], it is necessary to reconsider the risk of suicide due to affective reasons in schizophrenic subjects. However, the data in our possession are insufficient to clarify whether their parasuicidal behaviours are secondary to the presence of a major affective component or related to the presence of psychotic symptoms and their inadequate treatment.

#### Alcoholism and drug addiction

The results of this study indicate that 21% of the subjects had drunk alcohol at the time of their parasuicidal gesture and that about 18% had a history of a disturbance due to alcoholism. These results seem to be consistent with those of published studies showing a clear association between alcohol dependence and parasuicidal and suicidal behaviours [38]. Suokas and Lonnqvist [39] indicate that 62% of parasuicides are related to alcohol abuse at the time of the gesture or immediately beforehand. About 40% of the subjects being treated for a disorder due to alcohol dependence have a history of parasuicidal episodes, and about 5% of the subjects with such a disorder commit suicide [40,41]. It is also known that there is a significant association between alcoholism and depressive disorders, on the basis of which it is possible to hypothesise that the affective disorders may contribute towards determining the high incidence of suicidal behaviours among alcoholics [5].

Furthermore, various data indicate that substance abuse is also related to a high incidence of suicidal and parasuicidal behaviours [5,42], and we found that 8.5% of the patients in our sample had a history of diagnosed substance abuse.

Chronic substance abuse is often accompanied by a progressive loss of affective-relational ties, a worsening reduction in working function, and consequent social isolation. These conditions favour the onset of depressive experiences and a loss of self-esteem that increase the likelihood of adopting suicidal and parasuicidal behaviours [43]. Like alcohol, substances of abuse can facilitate parasuicidal gestures in various ways: they can be used because of their own self-injuring effects, as a means of self-disinhibition, or in order to increase the lethal nature of pharmaceutical drugs and alcohol.

It is therefore useful to underline the need for a wider ranging clinical and anamnestic evaluation of the subjects who are dependent on alcohol or substances of abuse that includes an investigation into the presence of psychic and socio-familial problems, as well as suicidal ideation.

#### Organic diseases

There are numerous published data showing that the presence of clinically relevant organic diseases is associated with a high parasuicidal and suicidal risk [5,44]. De Leo et al. [45] have pointed out that about half of the people who commit parasuicidal acts have a chronic organic disease. Forty-five percent of the subjects with a chronic disease consider it one of the factors precipitating their parasuicidal gesture, and 22% the determining factor [45]. In line with these observations, our results indicate that many (24.9%) of the subjects making parasuicidal gestures had chronic organic diseases (cardiovascular disturbances, oncological diseases, acquired immunodeficiency, diabetes mellitus) and, in comparison with the rest of the sample, were characterised by an older age (49.40 years ± SD 16.66) and a high frequency of previous parasuicidal episodes (46.5% of the cases).

Any disease affecting life expectancy is accompanied by inevitable changes in self-perception and socio-familial role. Maintaining the ability to project oneself into the future is a mental construct that is essential for preventing the onset of the ideation of death and the implementation of suicidal acts [46].

## Conclusion

In line with those of many previous studies, our results indicate that the patients at highest risk of parasuicide have a number of characteristic traits, including the presence of psychiatric disorders, organic diseases, alcohol/drug dependence/abuse, and significant psycho-relational disturbances.

However, it is precisely these characteristics that often hinder the creation of an optimal therapeutic alliance, and make these patients much less compliant to both pharmacological and psychotherapeutic treatment strategies.

In line with previous published data, we found that psychoactive drugs (particularly the benzodiazepines) are the most frequently used by people making parasuicidal gestures. Although not fatal (and regardless of the lethality of the drug used), parasuicidal behaviours are characterized by a poor prognosis insofar as they are frequently repetitive and lead to a high risk of death by suicide. It is therefore necessary to establish a therapeutic programme for such patients that covers both the acute situation and long-term prevention. In the case of patients who verbalize suicidal ideations and are being treated pharmacologically (particularly if they are receiving psychoactive drugs), it is essential to choose drugs that are less lethal if an excessive dose is taken.
